# Comparison of nested, multiplex, qPCR; FISH; SeptiFast and blood culture methods in detection and identification of bacteria and fungi in blood of patients with sepsis

**DOI:** 10.1186/s12866-014-0313-4

**Published:** 2014-12-11

**Authors:** Tomasz Gosiewski, Agnieszka Flis, Agnieszka Sroka, Anna Kędzierska, Agata Pietrzyk, Jolanta Kędzierska, Rafał Drwiła, Małgorzata Bulanda

**Affiliations:** Department of Bacteriology, Microbial Ecology and Parasitology, Chair of Microbiology Jagiellonian University Medical College, Czysta 18 Str., 31-121 Krakow, Poland; Department Epidemiology of Infection, Chair of Microbiology Jagiellonian University Medical College, Czysta 18 Str., 31-121 Krakow, Poland; Intensive Care Unit of the Cardiac Surgery Department, The John Paul II Hospital in Krakow, Pradnicka 80 Str., 31-202 Krakow, Poland; Department of Microbiology of the University Hospital in Krakow, Kopernika 19 Str., 31-501 Krakow, Poland; Department of Anesthesiology and Intensive Care, Jagiellonian University, Medical College, Kopernika 17 Str., 31-501 Krakow, Poland

**Keywords:** Nested, Multiplex qPCR, FISH, SeptiFast, Blood culture, Sepsis

## Abstract

**Background:**

Microbiological diagnosis of sepsis relies primarily on blood culture data. This study compares four diagnostic methods, i.e. those developed by us: nested, multiplex, qPCR (qPCR) and FISH with commercial methods: SeptiFast (Roche) (SF) and BacT/ALERT® 3D blood culture system (bioMérieux). Blood samples were derived from adult patients with clinical symptoms of sepsis, according to SIRS criteria, hospitalized in the Intensive Care Unit.

**Results:**

Using qPCR, FISH, SF, and culture, microbial presence was found in 71.8%, 29.6%, 25.3%, and 36.6% of samples, respectively. It was demonstrated that qPCR was significantly more likely to detect microorganisms than the remaining methods; qPCR confirmed the results obtained with the SF kit in all cases wherein bacteria were detected with simultaneous confirmation of Gram-typing. All data collected through the FISH method were corroborated by qPCR.

**Conclusions:**

The qPCR and FISH methods described in this study may constitute alternatives to blood culture and to the few existing commercial molecular assays since they enable the detection of the majority of microbial species, and the qPCR method allows their identification in a higher number of samples than the SF test. FISH made it possible to show the presence of microbes in a blood sample even before its culture.

## Background

Detecting the presence of microorganisms in the patient’s blood is crucial to validate the diagnosis of sepsis. Until now, the so-called diagnostic “gold standard” has been constituted by blood cultures carried out on special, universal growth media, preferably in automated cell culture systems. The advantages of such methods are their simplicity and relatively low costs of testing. Their weakness is that they are time-consuming, taking up to 5 days (until the test results are issued), and have low sensitivity, which causes only 15–20% of the culture to obtain microbial growth. Detecting microbes in blood is very difficult on account of their relatively small number, additionally limited by previously applied antibiotic treatment. [1,2]. Other, alternative, methods of detection of microbes in blood are being investigated [3-5]. These could reduce laboratory diagnosis time and provide greater sensitivity. An alternative is delivered by molecular biology, which enables precise and rapid detection of microbial genetic markers. Methods based on PCR techniques come to the forefront. Unfortunately, identification of microbes directly in blood encounters numerous obstacles associated with their very small number in the sample, the presence of inhibitors disrupting DNA amplification and the need to obtain nucleic acid isolates of very good quality [6,7]. The mentioned difficulties were the reason why, so far, there have been very few commercially available diagnostic kits for molecular diagnosis of sepsis, such as, SeptiFast (Roche), SeptiTest (Molzym), or VYOO (SIRS-Lab) [8]. As an alternative to nucleic acid amplification methods, FISH (Fluorescent In Situ Hybridization) could be employed. Up to now, it has been applied only with blood samples following culture, which entails the necessity to wait for multiplication of microorganisms [9,10]. In the literature, there are reports on the use of a method based on the detection of microbial proteins which allows to detect the species, i.e. MALDI-TOF MS (matrix-assisted laser desorption/ionization-time-of-flight mass spectrometry) and its commercial counterpart, Sepsityper (Bruker Daltonics), which, however, requires prior culture of the microorganism [11].

In blood infection treatment, the most important and most difficult issue determining the effectiveness of therapy and, consequently, the cost and duration of hospitalization, is the diagnosis of causes of infection. Rapid and modern diagnosis of sepsis requires application of methods that enable the detection of microorganisms directly from the patient’s blood sample within a few hours, which will increase the chances of the patient’s survival [12].

This study presents the results of laboratory tests on comparison of the efficacy of four methods for detecting the presence of microorganisms in the blood of adult patients with clinical symptoms of sepsis according to SIRS (systemic inflammatory response syndrome) criteria, i.e.: SeptiFast (Roche) (SF), nested, multiplex, qPCR (qPCR) [13], FISH [14] and blood culture using the BacT/ALERT 3D system (bioMérieux).

## Results

### Analysis of patients’ blood samples using methods: nested, multiplex, qPCR (qPCR), FISH, SeptiFast (SF) (Roche), and microbiological blood culture

71 blood samples taken from patients with clinical symptoms of sepsis were tested by applying the developed methods (qPCR and FISH) and a commercial SF kit and with the use of BacT/ALERT® 3D blood culture (bioMérieux). The application of qPCR enabled to increase the percentage of positive results to 71.8% in comparison with 36.6% in the case of culture. A lower percentage of positive results was also recorded for SF (25.3%) and FISH (29.6%) compared with the one obtained using qPCR (Table 1).

**Table 1:** **Comparison of the results obtained from blood of patients with clinical symptoms of sepsis by the method of blood culture, the nested multiplex qPCR, FISH and SeptiFast (Roche) methods and the internal inhibition control for the β-actin gene**

**(n = 71)**	**Blood culture**	**Nested multiplex qPCR**				**SeptiFast (Roche)**	**FISH**	**βactin gene**
		**Gram positive**	**Gram negative**	**Yeast**	**Filamentous fungi**			
1								+
2								+
3	*S. warneri*							+
4								+
5	*S. epidermidis*	+				CoNS	+	+
6			+					+
7	*S. haemolyticus*	+					+	+
8								+
9	*S. epidermidis*	+	+			CoNS	+	+
10								+
11			+					+
12			+					+
13	*E. cloacae*		+			*E. cloacae/aerogrenes*	+	+
14	*P. aeruginosa*		+				+	+
15	*S. haemolyticus*	+				*Staphylococcus. spp*	+	+
16			+					+
17			+					+
18	*S. hominis*	+					+	+
19			+					+
20								+
21			+			*E. cloacae/aerogrenes*		+
22		+						+
23		+						+
24			+					+
25			+			*E. cloacae/aerogrenes*	+	+
26								+
27			+					+
28	*S. haemolyticus*	+				CoNS	+	+
29								+
30			+			*E. cloacae/aerogrenes*		+
31			+					+
32			+			*K. pneumoniae/oxytoca*		+
33			+					+
34	*E. faecium*	+	+			*E. faecium*	+	+
35	*S. mitis*	+	+			*Streptococcus. spp*	+	+
36	*S. hominis*		+				+	+
37	*S. haemolyticus*							+
38			+					+
39	*P. aeruginosa*		+			*P. aeruginosa*	+	+
40			+					+
41			+			*A. fumigatus*		+
42			+					+
43			+					+
44	*S. epidermidis*							+
45			+					+
46			+					+
47	*C. albicans*					*C. albicans*		+
48								+
49			+			*K. pneumoniae/oxytoca*		+
50	*E. cloacae*							+
51								+
52								+
53	*S. hominis*	+	+					+
54		+	+			*S. aureus*		+
55								+
56			+					+
57		+	+					+
58		+	+					+
59	*K. pneumoniae*	+	+					+
60	*S. epidermidis*	+	+				+	+
61			+					+
62	*P. acnes*	+	+					+
63		+	+			*E. cloacae/aerogenes*	+	+
64		+	+				+	+
65	*P. aeruginosa* and *S. hominis*							+
66	*S. epidermidis*	+	+				+	+
67	*K. pneumoniae*	+	+			*K. pneumoniae/oxytoca*	+	+
68	*S. aureus*	+	+				+	+
69	*S. epidermidis*	**+**	+				+	+
70		+	+				+	+
71			+					+
**positive**	**26** *	**24**	**46**	**0**	**0**	**18** *	**21** *	**71**
		**51**						
**%**	**36.6**	**33.8**	**64.8**	**0**	**0**	**25.3**	**29.6**	**100**
		**71.8**						
**Sensitivity [CFU/ml]**	**1x10** ^**1**^ [1]	**1.1x10** ^**1**^ [13]	**1.3x10** ^**1**^ [13]	**8.5x10** ^**1**^ [13]	**3.7x10** ^**1**^ [13]	**3x10** ^**0**^ **– 3x10** ^**1**^ [28]	**6x10** ^**3**^ [14]	-

CoNS – coagulase negative *Staphylococcus.*

CFU = Colony Forming Unit.

„**+” –** positive result.

„*” – statistically significant differences in comparison with the nested, multiplex, qPCR (there was no significance between the other methods). Cochran’s Q test; p < 0.0001.

The bold data - summary of results.

The developed qPCR method enabled to corroborate the results obtained with the use of the SF kit in all cases in which bacteria were detected with simultaneous confirmation of Gram-typing. Two results for fungi were not supported (*C. albicans* and *A. fumigatus*).

**Figure 1:**
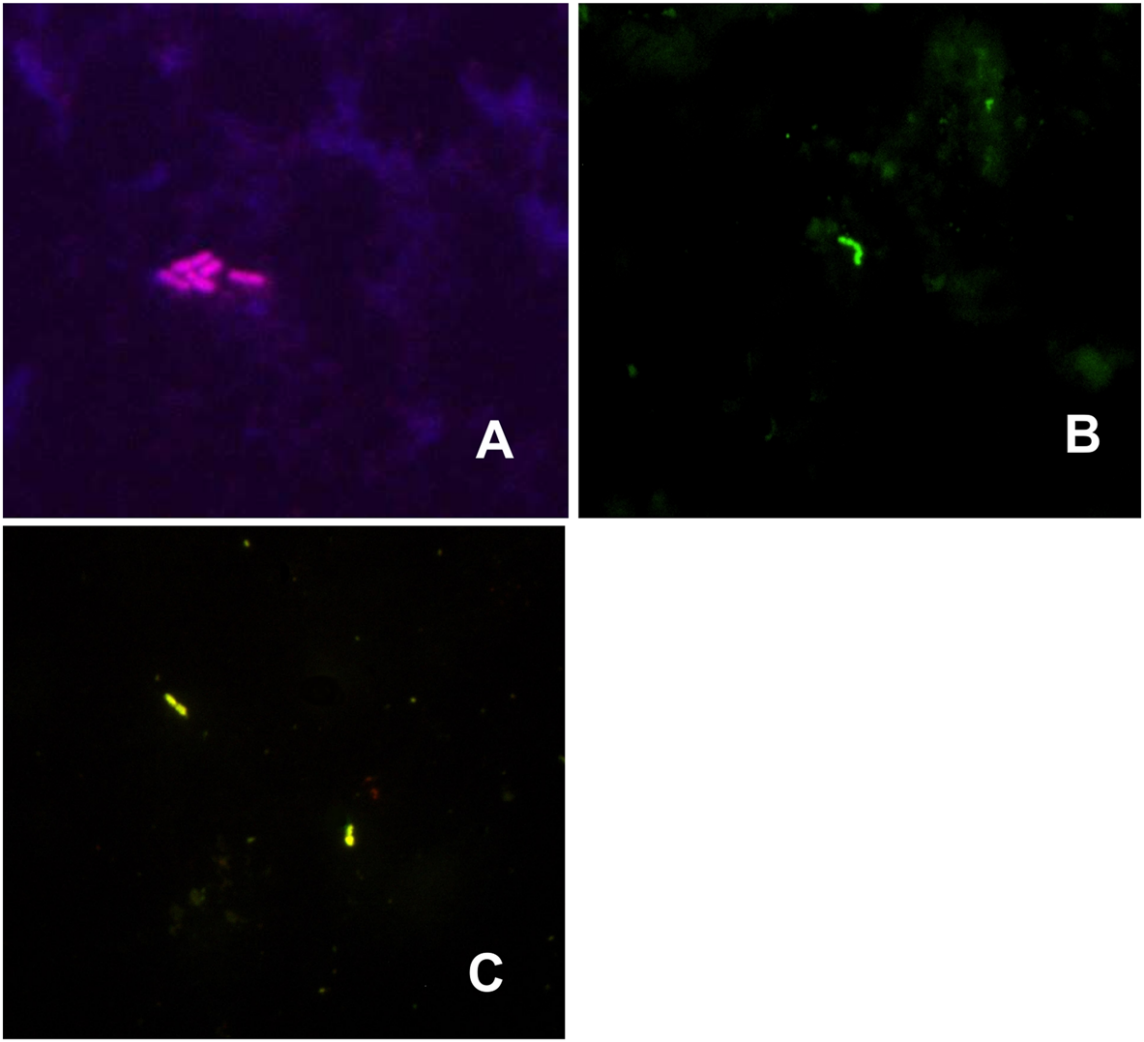
**Picture from a fluorescence microscope, obtained with the use of FISH: (A)** **visible** ***K. pneumoniae*** **bacteria (sample no. 67);** **(B)** **visible** ***Streptococcus*** **spp. bacteria (sample no. 35);** **(C)** **visible** ***E. cloacae*** ***bacteria*** **(sample no. 13).** Magnification 1000 x.

Neither qPCR nor SF confirmed positive results of blood culture in the same 6 samples (Table 1). All results gathered with the use of FISH (Figure 1) were confirmed by qPCR (Table 1).

It was demonstrated that qPCR shows the presence of microorganisms in blood significantly more frequently that culturing, FISH, or SF (p < 0.0001; Q = 52.15385). No significant differences between the remaining methods were determined.

In all 71 samples, amplification signal was obtained for the beta-actin gene, confirming that there was no inhibition of the amplification reaction. The applied negative controls in the form of DNA from sterile blood did not give an amplification signal.

## Discussion

An alternative to the classic diagnosis of sepsis by means of blood cultures is necessary. From the point of view of a physician and the quality of care of the patient with a blood infection, it is essential to get a microbiological confirmation of sepsis as quickly as possible. Unfortunately, blood cultures frequently require as long as several days of waiting for the results, and the outcome is often a false negative [15]. The methods developed by our team: nested, multiplex, qPCR [13] and FISH [14] were used to attempt to diagnose sepsis in blood samples of adult patients with its clinical symptoms and compared with the effectiveness of the commercial SeptiFast kit (Roche) and the BacT/ALERT 3D blood culture system (bioMérieux).

The percentage of positive results obtained using qPCR amounted to 71.8%, while the other methods, i.e. culture, FISH and SF, gave 36.2%, 29.6%, and 25.3%, respectively, and were significantly lower in comparison with qPCR (Table 1). qPCR enabled Gram-typing of bacteria, however, contrary to SF, did not make it possible to determine the species.

Such a high result for qPCR may suggest that there has been contamination, especially since nested amplification was employed, however, negative control was used in every case and it always gave a negative result. Moreover, we obtained very similar data during the testing of blood samples derived from children, in which qPCR allowed to achieve a proportion of positive results amounting to 69.6% [13]. Additionally, used primers specific to 16S rRNA sequences which could detect most bacteria species what could be the reason of high percentage of positive results. All blood samples came from patients with clinical symptoms of sepsis, which may suggest that, in the majority of cases, bacteremia or fungemia occurred, but it was impossible to detect them with the use of the remaining methods. The developed qPCR was based on 16S rDNA and 18S rDNA sequence-specific primers; hence, it allowed the identification of most species of bacteria and fungi, whereas the reference SF method enabled the detection of only over a dozen selected microbial species [16]. It is possible that the available methods of microbiological diagnostics of blood, i.e. culturing and sparse molecular tests, have limitations that only allow the detection of the most common, from the epidemiological point of view, microbial species. In patients recovering from extensive surgical procedures or not fully immunocompetent, bacterial translocation from the gastrointestinal tract, oral cavity, or from the outside may occur, which we are not able to confirm using commercially available diagnostic methods [17,18]. The application of nested PCR makes it possible to achieve greater sensitivity, which, in turn, allows considerable increase in the detection of bacteremia, which was showed by Benítez-Páez et al. whose proportion of positive results was 62.5% [19]. According to the researchers, the classic diagnostic methods enable marking of only several percent of bacterial species in the course of bacteremia, while the remaining portion is undetectable [19].

With the use of qPCR, all results obtained through the SF kit as regards bacteremia were confirmed, however, it failed for two samples, for which the SF test demonstrated the presence of *C. albicans* (also corroborated by culture) and *A. fumigatus*. The developed qPCR did not demonstrate the presence of fungi in any of the studied samples, which may have been caused by difficulties with fungal DNA isolation, probably due to their thick cell wall. On the other hand, fungemia occurs in patients following cardiac surgery in approx. 1% of cases [20]; hence, the examined group of patients may have been too small to assess the efficiency of qPCR as regards its capability to identify fungi in blood. The indication of the presence of *A. fumigatus* by the SF assay could have been due to contamination, since the occurrence of this species in blood is very rare and correlated mainly with patients with severe hematologic disorders [21]. Source of contamination was probably the hospital environment and the contamination might have occurred at the stage of a blood sample taking from the patient or during the investigation of samples in the laboratory.

Until now, the only description of the use of FISH for detection of bacteremia directly in a blood sample was the study presented by our team [14]; there are, however, numerous studies on the use of FISH for detecting bacteria in blood samples following culture [9,10,22]. The agreement between the traditional blood culture method and the PNA-FISH techniques was approximately 98% in each study, however, it was possible to detect the presence of bacteria in the culture which did not accelerate the process of microbiological diagnostics [9,10,22]. The FISH method made it possible to detect bacteria in 29.6% of samples and all the results were confirmed by qPCR. The SF method did not substantiate the results in 10 cases, which can be explained by the fact that, using FISH, EUB338 probe was used, which was specific for most bacterial species, while SF identified only over a dozen of them [16], in contrast to qPCR. FISH is cheaper and easier than SF, therefore, it is suitable as rapid screening of blood samples in patients with suspected sepsis.

The sensitivity of methods used to detection of microorganisms in the blood, expressed in CFU/ml units was presented in Table 1. According to Jamal and colleague blood culture method was able to detect microbial cells at level 1 × 10^2^ CFU/ml [1] as for Nested, multiplex, qPCR (Table 1) [13]. On the other hand, blood culture allowed to detect the presence of microorganisms in 36% of samples compared to 71% in the PCR method. Those difference probably results from the fact that the bacteria in the blood were inhibited by the immune system or used antibiotics which reduced the chances of their multiplication in a culture medium. PCR methods detected their DNA only.

## Conclusions

Molecular diagnosis of sepsis is becoming imperative on account of the fact that microbiological cultures are insufficient as regards their sensitivity and promptness. The qPCR and FISH methods, which were described in this study, may constitute an alternative to blood cultures and to the few commercially available molecular assays since they allow the detection of the majority of microbial species and qPCR enables their detection in a greater number of samples than the SF test. FISH made it possible to find microbes in a blood sample even before its culture.

## Methods

### Blood samples

71 blood samples were taken from patients with clinical symptoms of sepsis, hospitalized in the John Paul II Hospital in Krakow at The Ward of Anesthesiology and Intensive Care. Blood samples were drawn into 4-ml Vacutainer K_3_E (BectonDickinson) test tubes. Patients were enrolled into the study according to the SIRS criteria [23].

### Ethics statement and participants

The research was granted approval by the local Bioethics Committee of the Jagiellonian University (KBET/94/B/2009). Written informed consent was obtained from participants before their enrollment in the study.

### Blood culture

The blood culture was carried out in the John Paul II Hospital in Krakow in the Microbiology Department using BacT/ALERT® 3D apparatus (bioMérieux).

### Nested multiplex qPCR (qPCR) amplification

#### The method for microbial DNA isolation from blood

Microbial DNA was isolated from 1.5-ml blood samples according to the method described by Gosiewski et al. with the employment of a ready-to-use Blood Mini (A&A Biotechnology) [6].

#### DNA purity and concentration

The concentration and purity of total DNA isolates in the samples were measured spectrophotometrically at wavelengths of *A*_260_ and *A*_280_. It was performed in a NanoDrop machine (Thermo Scientific).

#### PCR amplification

All the processes of DNA amplification were performed with the use of the real-time PCR method (qPCR) in a CFX96 thermal cycler (BioRad) by employing species-specific primers and TaqMan probes (Genomed) according to procedure designed by Gosiewski et al. [13]. Additionally, in every sample of DNA isolated from blood, β-actin gene detection was performed in order to check whether rtPCR inhibition takes place; SYBR®Green JumpStart Taq ReadyMix (Sigma) was used for that purpose [24].

### FISH method

200 μl of blood was prepared according to procedure described by Gosiewski and colleagues using ammonium chloride solution (ICN Biomedicals), as in the case of preparing blood samples for DNA isolation, until a pale pink pellet was obtained [6]. The pellet was suspended in 20 μl of sterile deionized water from which 10 μl was transferred onto SuperFrost®Plus (Menzel–Glaser) microscope slide. The preparation was fixed with 500 μl of 4% paraformaldehyde (Sigma) solution for 20 min at 4°C. Then, the preparation was washed with PBS and poured over with 2 ml of 96% methanol (POCh) for 15 min at −20°C. Further, methanol was washed off with warm (37°C) PBS solution and placed on 20 μl of diluted solution of lysozyme (1 mg/ml) (Sigma) and lysostaphin (0.05 mg/ml) (Sigma) for 5 min at 37°C. Hybridization was performed with the use of probes (Genomed) labeled with fluorochromes at 5′ ends, targeted at the 16S rRNA: *Staphylococcus* STA probe was used (CY3-5′ – TCC TCC ATA TCT CTG CGC 3′) [25]; *Enterobacteriaceae,* ENT183 probe (CY3-5′ – CTC TTT GGT CTT GCG ACG- 3′) [26]; for all bacteria, EUB338 probe was used (FITC-5′ – GCT GCC TCC CGT AGG AGT - 3′-FITC) [27]. 5 μl of EUB338 and STA or ENT183 probes solution (50 ng/μl) was mixed with 40 μl of hybridization buffer: 20 mM Tris HCl pH 7.2 (Sigma); 0.9 M NaCl (Serva); 0,1% SDS (Serva) heated to 50°C. The resulting solution was transferred onto the preparation and placed at 50°C in a humid chamber for 2 h. Afterwards, the probe was washed off with warm hybridization buffer, except SDS and the preparation was stained with DAPI (4′,6-diamidino-2-phenylindole) (Sigma) 15 μg/ml for 3 min. The stained preparation was thoroughly washed with sterile distilled water and dried in the dark. The specimen was viewed using BX51 fluorescence microscope (Olympus) and F-View camera (Olympus). The resulting image was analyzed using AnalySYS (Soft Imaging) software.

### SeptiFast (Roche) analysis

DNA isolation, PCR amplification, and analysis of the resulting data were conducted in the Department of Microbiology of the University Hospital in Kraków, according to the protocol supplied by the manufacturer and with the use of Roche software.

### Statistics

The discrepancies between the methods as regards their ability to detect microorganisms was studied using Cochran’s Q test (Gretl software ver. 1.9.4.). *P* value of <0.05 was taken as statistically significant.

## References

[1] Jamal W, Tamaray G, Pazhoor A, Rotimi VO (2006). Comparative evaluation of BacT/ALERT 3D and BACTEC systems for the recovery of pathogens causing bloodstream infections. Med Princ Pract.

[2] Loonen AJM, Wolffs PFG, Bruggeman CA, van den Brule AJC (2014). Developments for improved diagnosis of bacterial bloodstream infections. Eur J Clin Microbiol Infect Dis.

[3] Burdino E, Ruggiero T, Allice T, Milia MG, Gregori G, Milano R, Cerutti F, De Rosa FG, Manno E, Caramello P, Di Perri G, Ghisetti V (2014). Combination of conventional blood cultures and the SeptiFast molecular test in patients with suspected sepsis for the identification of bloodstream pathogens. Diagn Microbiol Infect Dis.

[4] Schreiber J, Nierhaus A, Braune SA, de Heer G, Kluge S (2013). Comparison of three different commercial PCR assays for the detection of pathogens in critically ill sepsis patients. Med Klin Intensivmed Notfmed.

[5] Leitner E, Kessler HH (2015). Broad-range PCR for the identification of bacterial and fungal pathogens from blood: a sequencing approach. Methods Mol Biol.

[6] Gosiewski T, Szała L, Pietrzyk A, Brzychczy-Włoch M, Heczko PB, Bulanda M (2014). Comparison of methods for isolation of bacterial and fungal DNA from human blood. Curr Microbiol.

[7] Schrader C, Schielke A, Ellerbroek L, Johne R (2012). PCR inhibitors - occurrence, properties and removal. J Appl Microbiol.

[8] Skvarc M, Stubljar D, Rogina P, Kaasch AJ (2013). Non-culture-based methods to diagnose bloodstream infection: Does it work?. Eur J Microbiol Immunol (Bp).

[9] Calderaro A, Martinelli M, Motta F, Larini S, Arcangeletti MC, Medici MC, Chezzi C, De Conto F (2013). Comparison of peptide nucleic acid fluorescence in situ hybridization assays with culture-based matrix-assisted laser desorption/ionization-time of flight mass spectrometry for the identification of bacteria and yeasts from blood cultures and cerebrospina. Clin Microbiol Infect.

[10] Farina C, Perin S, Andreoni S, Conte M, Fazii P, Lombardi G, Manso E, Morazzoni C, Sanna S (2012). Evaluation of the peptide nucleic acid fluorescence in situ hybridisation technology for yeast identification directly from positive blood cultures: an Italian experience. Mycoses.

[11] Schieffer KM, Tan KE, Stamper PD, Somogyi A, Andrea SB, Wakefield T, Romagnoli M, Chapin KC, Wolk DM, Carroll KC (2014). Multicenter evaluation of the Sepsityper^TM^ extraction kit and MALDI-TOF MS for direct identification of positive blood culture isolates using the BD BACTEC^TM^ FX and VersaTREK(®) diagnostic blood culture systems. J Appl Microbiol.

[12] Daniels R (2011). Surviving the first hours in sepsis: getting the basics right (an intensivist’s perspective). J Antimicrob Chemother.

[13] Gosiewski T, Jurkiewicz-Badacz D, Sroka A, Brzychczy-Włoch M, Bulanda M (2014). A novel, nested, multiplex, real-time PCR for detection of bacteria and fungi in blood. BMC Microbiol.

[14] Gosiewski T, Pietrzyk A, Brzychczy-Wloch M, Heczko P (2011). Use of PCR and FISH methods for rapid identification of bacterial bloodstream infections. Ann Acad Med Siles.

[15] Liesenfeld O, Lehman L, Hunfeld K-P, Kost G (2014). Molecular diagnosis of sepsis: new aspects and recent developments. Eur J Microbiol Immunol (Bp).

[16] Chang S-S, Hsieh W-H, Liu T-S, Lee S-H, Wang C-H, Chou H-C, Yeo YH, Tseng C-P, Lee C-C (2013). Multiplex PCR system for rapid detection of pathogens in patients with presumed sepsis - a systemic review and meta-analysis. PLoS One.

[17] Aboelatta YA, Abd-Elsalam AM, Omar AH, Abdelaal MM, Farid AM (2013). Selective digestive decontamination (SDD) as a tool in the management of bacterial translocation following major burns. Ann Burns Fire Disasters.

[18] Samet A, Sledzińska A, Krawczyk B, Hellmann A, Nowicki S, Kur J, Nowicki B (2013). Leukemia and risk of recurrent Escherichia coli bacteremia: genotyping implicates E. coli translocation from the colon to the bloodstream. Eur J Clin Microbiol Infect Dis.

[19] Benítez-Páez A, Álvarez M, Belda-Ferre P, Rubido S, Mira A, Tomás I (2013). Detection of transient bacteraemia following dental extractions by 16S rDNA pyrosequencing: a pilot study. PLoS One.

[20] Pasero D, De Rosa FG, Rana NK, Fossati L, Davi A, Rinaldi M, Di Perri G, Ranieri VM (2011). Candidemia after cardiac surgery in the intensive care unit: an observational study. Interact Cardiovasc Thorac Surg.

[21] Mancini N, Clerici D, Diotti R, Perotti M, Ghidoli N, De Marco D, Pizzorno B, Emrich T, Burioni R, Ciceri F, Clementi M (2008). Molecular diagnosis of sepsis in neutropenic patients with haematological malignancies. J Med Microbiol.

[22] Parcell BJ, Orange GV (2013). PNA-FISH assays for early targeted bacteraemia treatment. J Microbiol Methods.

[23] Levy MM, Fink MP, Marshall JC, Abraham E, Angus D, Cook D, Cohen J, Opal SM, Vincent J-L, Ramsay G (2001). SCCM/ESICM/ACCP/ATS/SIS international sepsis definitions conference. Intensive Care Med.

[24] Valle DL, Andrade JI, Cabrera EC, Rivera WL (2010). Evaluation of buffy coat 16S rRNA PCR, buffy coat culture and whole blood PCR for detection of bacteraemia. Mem Inst Oswaldo Cruz.

[25] Kempf VA, Trebesius K, Autenrieth IB (2000). Fluorescent In situ hybridization allows rapid identification of microorganisms in blood cultures. J Clin Microbiol.

[26] Friedrich U, Van Langenhove H, Altendorf K, Lipski A (2003). Microbial community and physicochemical analysis of an industrial waste gas biofilter and design of 16S rRNA-targeting oligonucleotide probes. Environ Microbiol.

[27] Amann RI, Binder BJ, Olson RJ, Chisholm SW, Devereux R, Stahl DA (1990). Combination of 16S rRNA-targeted oligonucleotide probes with flow cytometry for analyzing mixed microbial populations. Appl Environ Microbiol.

[28] Westh H, Lisby G, Breysse F, Böddinghaus B, Chomarat M, Gant V, Goglio A, Raglio A, Schuster H, Stuber F, Wissing H, Hoeft A (2009). Multiplex real-time PCR and blood culture for identification of bloodstream pathogens in patients with suspected sepsis. Clin Microbiol Infect.

